# Inhalational injury and use of heparin & N-acetylcysteine nebulization: A case report

**DOI:** 10.1016/j.rmcr.2022.101640

**Published:** 2022-03-22

**Authors:** Nissar Shaikh, Arshad H. Chanda, Mohammad A. Rahman, Mohammed M. Nainthramveetil, Ashish Kumar, Ranjan M. Mathias, Abdulqadir J. Nashwan

**Affiliations:** a Surgical Intensive Care Department, Hamad General Hospital (HGH), Hamad Medical Corporation (HMC), Doha, Qatar; b Nursing Department, Hazm Mebaireek General Hospital (HMGH), Hamad Medical Corporation (HMC), Doha, Qatar

**Keywords:** Burn injury, Inhalational injury, Tracheobronchial injury, Unfractionated heparin, N-acetylcysteine (NAC), UFH, unfractionated heparin, NAC, N-acetylcysteine, SICU, surgical intensive care unit, ETT, endotracheal tube

## Abstract

Inhalational injury to the upper and lower airway occurs due to thermal or chemical irritation causing airway edema, capillary leak, mucin, and fibrin debris forming clots and soot. The use of unfractionated heparin (UFH) nebulization was found to be effective by dissolving airway clots. We report a case of inhalational burn injury where UFH nebulization led to a better outcome. A healthy male was trapped in a residential room during a fire in the building. He sustained facial, neck, upper chest, and left upper extremity burns accounting for 25% of body surface area. He was intubated at the site and started on supportive care. In the surgical intensive care unit, bronchoscopy showed severe tracheobronchial burn injury; a thorough lavage was done, started on UFH and N-acetylcysteine nebulization (NAC). The patient improved, and his trachea was extubated on day 6. In our patient, unfractionated heparin nebulization was beneficial as the patient was extubated early without landing to acute respiratory distress syndrome.

## 1 Introduction

Inhalational injury can be caused by thermal injury or chemical irritation resulting in tracheobronchial epithelial injuries [1]. The inhalation injury due to fire smoke in burn patients remained a leading cause of morbidity and mortality [2]. Inhalational injury in burn patients was found to be an independent predictor of mortality [3]. In some reports, the management of inhalational injury, mostly conservative and supportive, unfractionated heparin (UFH) nebulization in combination with N-acetylcysteine (NAC) showed a better outcome. At the same time, it did not in other reports. We report a case of inhalational burn injury and the use of heparin and N-acetylcysteine (NAC) nebulization with a better outcome.

## 2 Case presentation

A 60-year-old male with no comorbidities was trapped in his room for an unknown period when fire brooked out due to a short circuit in the residential building. He sustained facial, neck, upper chest, and left upper extremity burns accounting for 25% of body surface area signs of inhalational injury. The patient was intubated by the emergency medical services on the site and found burn injury involving the nose and nasopharynx.

**Fig. 1:**
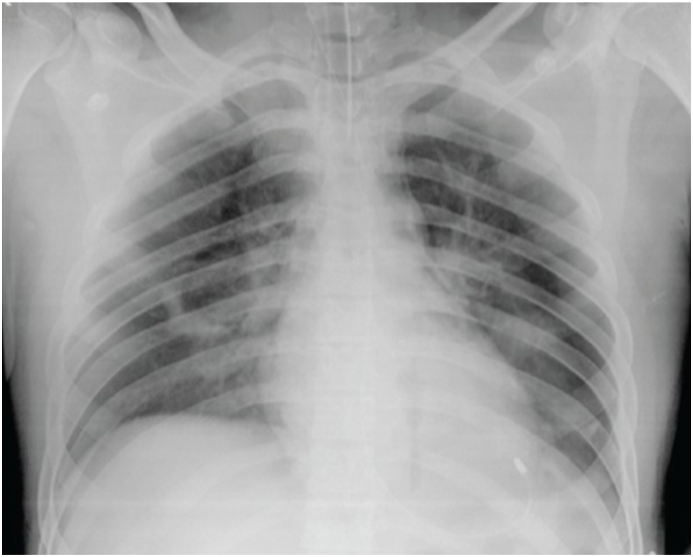
Shows Hilar and a few scattered infiltrates.

The patient was admitted to the surgical intensive care unit (SICU) through the emergency. He was sedated with propofol, and analgesia was supplemented with remifentanil. He was ventilated with 100% oxygen supplementation. Fluid resuscitation was continued with Evan's formula. His carbon monoxide levels were within normal range, and chest x-ray showed hilar and few scatted infiltrates (Fig. 1).

**Fig. 2:**
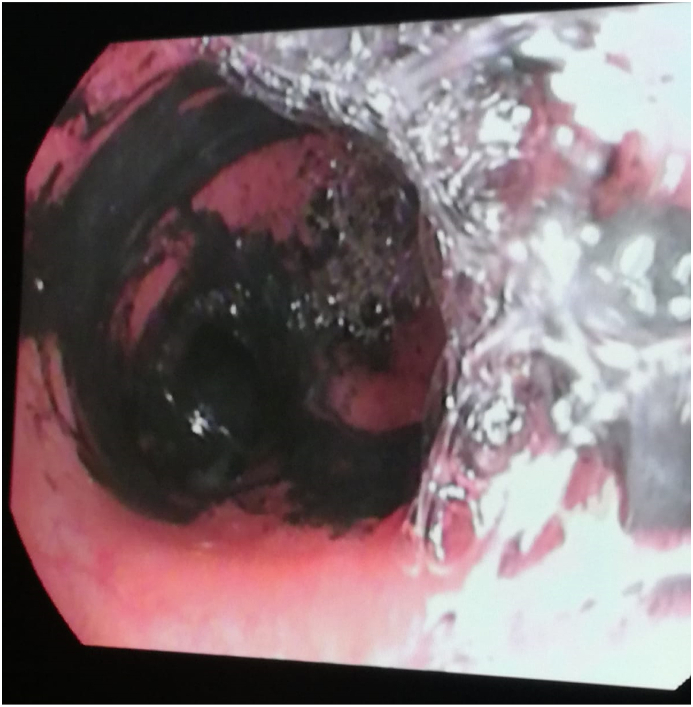
Bronchoscopic view showing dense soot (Melanoptysis).

In view of inhalational injury, fiberoptic bronchoscopy was performed; it showed severe tracheobronchial injury, edema, a lot of soot deposition, and melanoptysis (Fig. 2). Extensive irrigation and bronchoalveolar lavage were done. He was started on 10,000 IU unfractionated heparin (UFH) and 5ml of 10% N-acetylcysteine (NAC) nebulization every 6 hours and a vibrating mesh nebulizer synchronized with mechanical ventilation. He required a smaller dose of noradrenaline to maintain his hemodynamics. The patient was tolerating enteral feeds.

His hemodynamics and respiratory parameters were improving (Table 1), noradrenaline was weaned off, and he could breathe spontaneously by day 4. Repeat bronchoscopy also showed improvement in tracheobronchial injuries. UFH nebulization was stopped. He received a total of 120,000 units of UFH over 4 days, and there was no systemic anticoagulation or other side effects. On day 5, the patient underwent shaving and split-thickness skin graft for upper extremity burns.

**Table 1:** Showing improvement in ventilator and oxygenation parameters.

Day of admission	Fraction inspired oxygen	Respiratory Rate/minute	VT (Tidal Volume in Milliliters)	Pco2 (Partial pressure of oxygen in mm of Hg)	PEEP (Positive End Expiratory pressure)	Po2(Partial pressure of oxygen in mm of Hg)
1	1	20	430	47	10	71
2	0.6	18	450	42	8	92
3	0.5	16	450	39	6	96
4	0.4	16	460	38	6	103

The patient was awake and stable after the spontaneous breathing trial; his trachea was extubated successfully on day 6. All invasive lines were removed and started on a high protein oral diet, and he was ambulated. He was transferred to the burn unit on day 8 from there discharged home to be followed up in the outpatient clinic.

## 3 Discussion

Smoke inhalational injury is a concern in burn patients as it increases morbidity and mortality [[1], [2], [3]]. Inhalational injury occurs in up to 20% of burn patients [1]. The inhalational injury should be suspected in patients involved in burns and trapped in a close compartment; an initial imaging study will support the proper diagnosis, and bronchoscopy has a diagnostic and therapeutic value [4]. The management of the inhalational injury remains supportive. These patients are frequently intubated with a possible large-sized endotracheal tube (ETT) to facilitate the copious secretions and airway debris; it is vital to secure the ETT, as reintubation will be difficult [5].

The culprit of inhalational upper and lower airway injuries is thermal injury and chemical irritation. The thermal injury usually involves glottic and supraglottic structures causing massive swelling and edema leading to airway collapse or compromise. The lower airway is injured by the chemicals generated by burning physical structure rubber, plastics, and laminations [6]. These chemicals and toxins cause injury to the tracheobronchial and vascular endothelium, causing severe damage to the mucosa and loss of surfactant [6].

Combined with mucin, fibrin, and leaked plasma, these cellular debris forms clots and soot deposits [6]. All these factors mentioned above cause ventilation-perfusion mismatch and increased risk for consolidation and collapse.

Several therapies were explored to minimize the above-mentioned epithelial injuries and improve oxygenation in inhalational injury patients. Use of inhalational activated protein c, antithrombin, tissue pathway inhibitors, and plasminogen activator showed different levels of success in experimental studies [1].

The use of nebulized unfractionated heparin(UFH) and N-acetylcysteine (NAC) in inhalational injury was useful in both animal and human studies [7]. These agents inhibit clot formation and help in mucolysis, which has antioxidant and anti-inflammatory actions.

A study by McIntire et al., revealed that the UFH nebulization within 48 hours of inhalational injury, in the dose of 10,000 IU every 4 hourly for 7 days or till extubation in combination with N-acetylcysteine (NAC), had a significant reduction in duration of mechanical ventilation, and ventilator-free days [8]. Desai et al. showed in the pediatric population that the use of UFH 5000 IU and N-acetylcysteine (NAC) decreased reintubation rate, mortality, and development of pneumonia in inhalation injury patients [9]. In their review of inhalational injury found that the use of aerosolized UFH/N-acetylcysteine therapy increases survival and lowers morbidity without affecting systemic clotting and anticoagulation markers [10]. Kashefi et al. in their small powered retrospective study did not find any beneficial effect of heparin and NAC nebulization in inhalational burn injury patients [11]. Elsharnouby et al. compared different dosages of nebulized heparin in inhalational injury in their small powered study and found that 10,000 IU dose of UFH nebulization was more effective than 5000 IU dosage [12].

## 4 Conclusion

The use of aerosolized UFH and N-acetylcysteine in our patient who had an inhalational injury was effective and led to better outcomes by reducing the duration of mechanical ventilation and shortening the ventilator-free days in the ICU.

## Key learning points

Use of aerosolized UFH and N-acetylcysteine in inhalational burn injury may lead to

• Early liberation from mechanical ventilation
• Increase ventilator-free days
• Improved overall outcome

## Ethics approval and consent to participate

The article describes a case report. Therefore, no additional permission from our Ethics Committee was required (MRC-04-20-369).

## Consent for publication

The consent for publication was obtained from the patients.

## Availability of data and material

All data generated or analyzed during this study are included in this published article.

## Funding

This study was not funded.

## Authors' contributions

Data Collection: NSH.

Literature Search: NSH, ACH, MAR, MMN, AKU, RAM, AJN.

Manuscript Preparation (draft and final editing): NSH, ACH, RMA, MMN, AKU, RAM, AJN.

All authors read and approved the final manuscript.

## Declaration of competing interest

The authors declare that they have no known competing financial interests or personal relationships that could have appeared to influence the work reported in this paper.
